# 16S rRNA gene-based assessment of common broiler chicken sampling methods: Evaluating intra-flock sample size, cecal pair similarity, and cloacal swab similarity to other alimentary tract locations

**DOI:** 10.3389/fphys.2022.996654

**Published:** 2022-10-19

**Authors:** Margaret D. Weinroth, Brian Oakley, Gustavo A. Ramírez, Arquimides Reyes, Caitlin E. Harris, R. Jeff Buhr

**Affiliations:** ^1^ PMSPRU, USNPRC, USDA-ARS, Athens, GA, United States; ^2^ College of Veterinary Medicine, Western University of Health Sciences, Pomona, CA, United States; ^3^ Department of Biological Sciences, California State University, Los Angeles, CA, United States; ^4^ Department of Animal and Food Science, University of Wisconsin-River Falls, River Falls, WI, United States; ^5^ Department of Poultry Science, The University of Georgia, Athens, GA, United States

**Keywords:** 16S, microbiome, poultry (chicken), cecal microflora, cloacal swab

## Abstract

16S rRNA gene sequencing for characterization of microbiomes has become more common in poultry research and can be used to both answer specific research questions and help inform experimental design choices. The objective of this study was to use 16S rRNA gene sequencing to examine common sampling practices in broiler chicken studies such as: the required number of birds selected from a flock to adequately capture microbiome diversity, the differences between cecal pairs within the same bird, and whether cloacal swabs are representative of other alimentary tract (AT) locations. To do this, nine market age broilers were euthanized and immediately sampled in ten AT locations: crop, gizzard, proventriculus, duodenum, jejunum, ileum, cecal samples from each pouch, colon, and cloacal swab. DNA was extracted and subjected to 16S rRNA gene amplification and sequencing. Each location within the broiler AT hosts distinct microbial communities. When each sampling location was considered, it was found that sampling after 2.8 birds (range 2–4) resulted in less than 10% new amplicon sequencing variants (ASV) being added while sampling after 7.6 birds (range 6–10) increases new observed ASVs by less than 1%. Additionally, when cecal pairs from the same bird were evaluated, it was found that cecal pair mates are an adequate replication if interested in the total cecal microbiome but may be less useful if a rare lineage is of interest. Furthermore, when compared to other AT locations, the cecal microbiome was enriched in Firmicutes and Bacteroides while several lineages, most notably Lactobacillus, were under-represented. Finally, when cloacal swabs were compared to other AT locations, community similarity exhibited a direct distance relationship, i.e., the more aborad samples were the more similar they were to the swab. These findings indicate that while cloacal swabs can approximate overall changes in microbiome composition, they are not adequate for inferring changes to specific taxa in other parts of the AT tract—even those that are highly abundant within the microbial community. These data provide new insights guiding appropriate sample size selection within flocks and add to the consensus data regarding cecal pair similarity and destructive versus non-destructive sampling methods.

## Introduction

Next generation sequencing in poultry science has seen wider implementation as a research tool as sequencing costs fall and bioinformatic tools become more accessible (Clavijo and Flórez, 2018; Weinroth et al., 2022). Specifically, 16S rRNA gene sequencing has been used to survey the microbiome of many poultry and related environments (Bucher et al., 2020; Marmion et al., 2021; Yaqoob et al., 2021). Using a culture-free approach to survey a microbial community allows for the identification of both culturable and non-culturable microorganisms. Inevitably, there are also limitations to this approach, such as the inability to distinguish between live and dead cells, the use of relative abundance data (as opposed to absolute numbers such as enumeration of bacterial colonies on a plate), and difficultly in identifying less abundant bacterial taxa (Mira Miralles et al., 2019; Weinroth et al., 2022).

Previous broiler microbiome work has addressed descriptions of diversity within ceca (Mancabelli et al., 2016), modulation of the microbiome as the result of antibiotic and probiotic treatments (Gao et al., 2017), changes associated with age (Oakley et al., 2014) and season (Oakley et al., 2018), as well as the description of other broiler related microbiomes such as litter (Bucher et al., 2020), feed, and meat (Marmion et al., 2021). In broiler sampling, the choice to use lethal (requiring euthanasia of the bird such as a cecal sample) or non-lethal sampling (such as a cloacal swab) is also of interest. 16S rRNA gene sequencing has also been used to understand this challenge, through assessing the validity of non-lethal sampling techniques as a proxy for other alimentary tract (AT) locations and comparisons of two paired ceca within one bird (Andreani et al., 2020; Williams and Athrey, 2020). As sequencing capabilities and our understanding of the broiler associated microbiome continue to grow, the validation of sound sampling practices is of paramount importance. Here, by surveying nine alimentary tract (AT) sites and cloacal swabs within the same nine broiler chickens from a single flock, we aimed to assess the suitability of common sampling practices. The objectives of this study were to use 16S rRNA gene sequencing to examine common sampling practices in broiler studies such as the number of birds needed from a flock to capture the microbiome diversity, the differences between the paired cecal communities within single birds, identification of bacterial lineages that are enriched in the ceca relative to other parts of the AT, and the validity of using cloacal swabs as proxies for inferring microbial communities at other gastrointestinal tract locations.

## Materials and methods

Nine market age male broilers Cobb 500 were obtained full fed at flock termination from the University of Georgia Poultry Research Center, cooped, and transported to the US National Poultry Research Center (USNPRC) pilot processing plant where there they were individually euthanized following the USNPRC IACUC SOP#10 Euthanasia Methods approved for poultry (C. Electrocution of Poultry). Upon death, cloacal swabs were collected from each bird using a sterile PurFlock ultra regular tip double swabs (Puritan Medical Products, Guilford, ME) and kept on ice until storage at −20°C. For all birds the intact alimentary tract was excised and both the esophagus and vent were clamped to prevent ingesta leakage; all tracts were kept on ice until processing. For each tract, nine sampling locations were chosen in addition to the cloacal swab: 1) crop, 2) gizzard, 3) proventriculus, 4) duodenum, 5) jejunum, 6) ileum, (7–8) cecal samples from each pouch, and 9) large intestine (colon). For each sample, the outside was swabbed with alcohol and allowed to air dry. From there, a new sterile scalpel was used to open the location in the middle of the segment of interest cutting only one time as to not reintroduce outside contaminants. PurFlock double tip swabs were used to swab the inside of the tract with minimal pressure to capture the lumen microbiome. After sampling, swabs were immediately placed at −20°C until DNA extraction.

DNA was extracted from all samples in addition to two unused swabs to act as negative controls using a QIAGEN QICUBE HT with DNeasy 96 PowerSoil Pro QIAcube HT Kit following the manufacturer’s protocol (QIAGEN, Hilden, Germany). DNA was quantified using a Quantus fluorometer (Promega Corporation, Madison, WI). DNA (≥200 ng) was shipped to Novogene Corporation (Beijing, China) for library preparation with the V4 515/804R 16S rRNA primers and sequencing (2 × 250 bp) on an Illumina HiSeq (Illumina, Inc., San Diego, CA, United States) to a target depth of 30,000 reads per sample for all non-negative controls.

Demultiplexed samples were imported into QIIME2 v. 2021.8 (Bolyen et al., 2019) and amplicon sequencing variants (ASV) were assigned with DADA2 (Callahan et al., 2016) with the first 19 nucleotides of forward reads and the 20 lead nucleotides of the reversed reads trimmed as well as both reads truncation at 200. A phylogenetic tree was constructed with MAFFT v. 7 (Katoh and Standley, 2013) and FastTree2 (Price et al., 2010) while taxonomic classification was conducted using a Naïve Bayesian classifier pretrained using the 515-806R primers on the Greengenes database (DeSantis et al., 2006). Reads that were assigned to chloroplasts and mitochondria and those that did not have a kingdom classification were removed. Data was imported into R (4.0.2) using qiime2R (v0.99.6). Decontam (Davis et al., 2018) was used with the “combo” choice to remove contamination using fluorometer data and sequenced negative controls as well as the removal of two species of Lactobacillus that were high in negative controls but not known to be highly abundance in the chicken AT were removed (classified as *L. acidipiscis* and *L. helveticus*). The resulting ASV table was normalized using cumulative sum scaling (CSS) (Paulson et al., 2013).

Alpha diversity was measured as Shannon’s diversity (Shannon, 1948) in phyloseq (v. 1.34.0) (McMurdie and Holmes, 2013). Beta diversity was assessed with PCoA using Bray-Curtis dissimilarity and compared with ANOSIM also in vegan (v. 2.5-7) (Oksanen et al., 2014). ANCOM (Mandal et al., 2015) was used to compare family level relative abundance differences between AT locations and specifically between cecal pairs using the QIIME2’s composition plugin after the addition of a pseudo count. Family level differences between locations in the AT were compared with ordination and visualized with ggordiplots (0.4.1). Ordination of cecal pairs was conducted in the same way. Bray Curtis dissimilarity and weighted Unifrac distances were computed with phyloseq and visualized using ggplot2. Number of new and unique ASV were adapted from ROARY’s create_pan_genome_plots R script (Page et al., 2015 Roary: rapid large-scale prokaryote pan genome analysis | Bioinformatics | Oxford Academic) and were visualized in ggplot2. Differential enrichment analysis was performed using a Wald Test (*p* = 0.01) implemented on DESeq2 (Love et al., 2014). Phylogenetic comparison of differentially enriched lineages was conducted using the phyloseq plot_tree() function. Enterobacteriaceae normalized read correlations were compared and visualized in ggpubr 0.4.0. Alpha was set at 0.05.

## Results

Across all non-negative control samples, 7,282,584 reads were analyzed (average = 81,826, range 31,246–18,324) representing 45 phyla, 1,140 species, and 9,212 ASV.

### Modulation of the microbiome throughout the chicken alimentary tract

**FIGURE 1:**
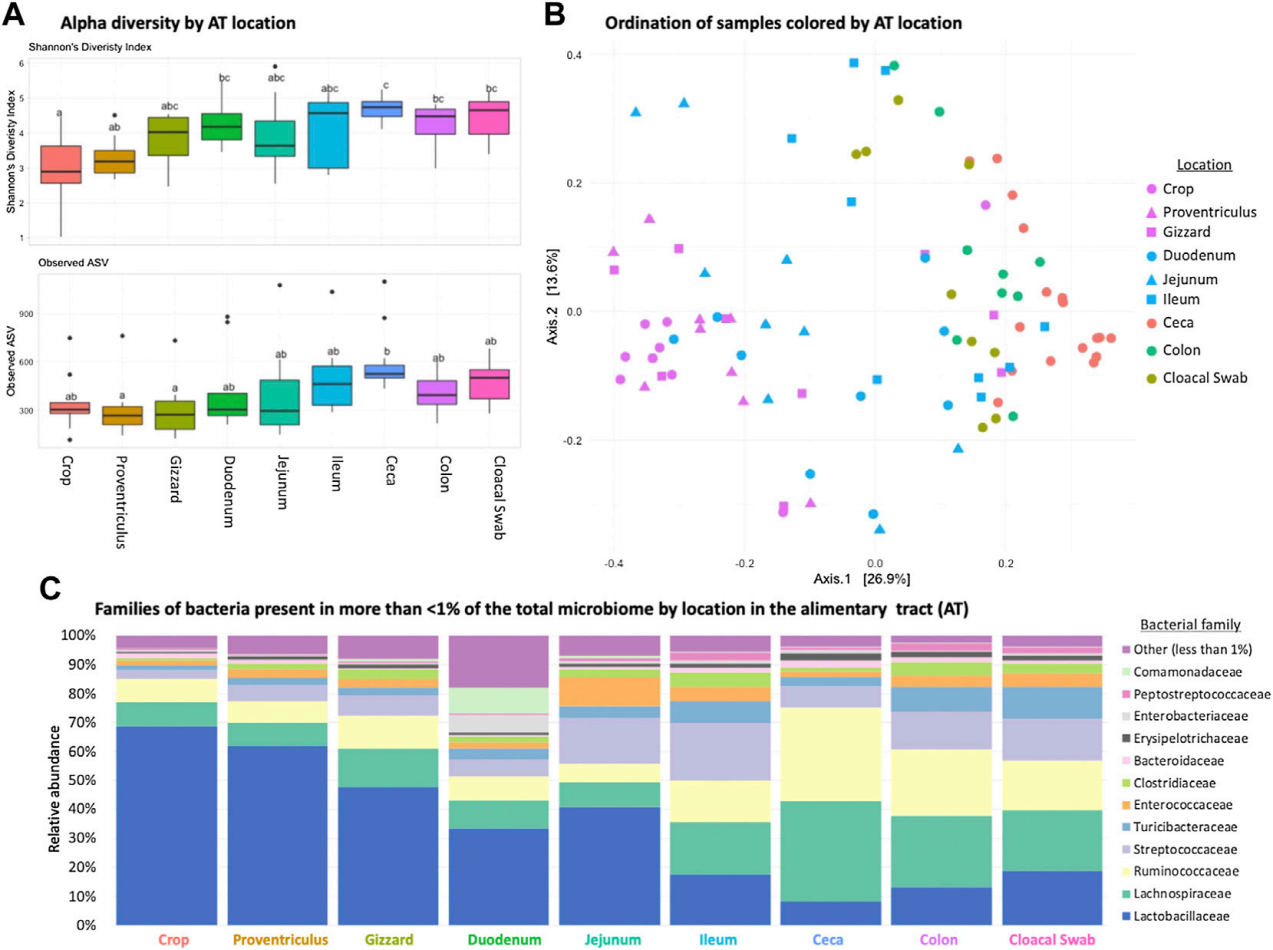
Differences in the broiler chicken alimentary tract (AT) by location. **(A)** Alpha diversity of AT locations in the birds; there were differences (*p* < 0.05) between locations. **(B)** Beta diversity differences of AT locations; locations were different (ANOSIM R = 0.6188, *p* = 0.001). **(C)** Stacked bar chart of relative abundance of more prominent bacterial classes found in the AT by location.

Each AT location sampled had a unique microbiome composition, with differences in alpha and beta diversity as well as the relative abundance of bacterial families (Figures 1A–C). Alpha diversity was lower (*p* < 0.05) in the orad portions of the AT and was numerically highest in the ceca (Figure 1A). When beta diversity was compared there were also differences according to AT location (ANOSIM R = 0.6188, *p* = 0.001, Figure 1B). Within all AT locations, Firmicutes were the most abundant phyla present with *Lactobacillaceae*, *Lachnospiraceae*, and *Ruminococcacceae* dominating family-level relative abundance (Figure 1C). Throughout the AT, *Lactobacillaceae* and *Lachnospiraceae* decreased in relative abundance from crop to colon.

### Within flock clonality to determine appropriate sample size

**FIGURE 2:**
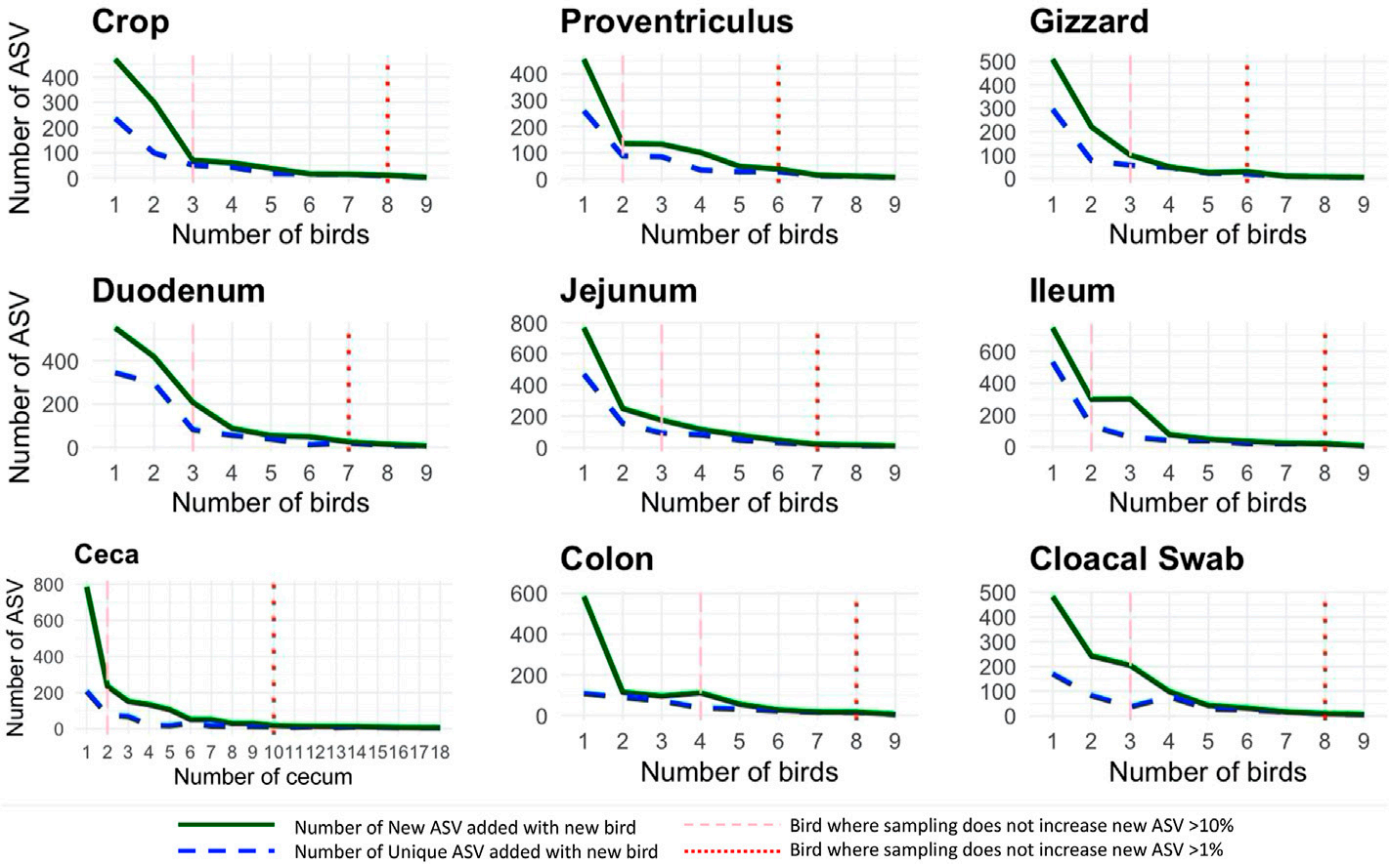
Number of new and unique amplicon sequence variants (ASVs) added when a new bird replication was added (faceted by AT location) with two different thresholds: the number of new ASVs increasing by <10% and <1%.

Throughout the AT, similarity of each location across birds was high as specific sites were similar in relative abundance of bacteria. When each AT location was assessed for the number of new ASVs added with the addition of a new bird as well as the number of unique ASV (those specific to just that bird), the same diminishing returns were found across all sample locations (Figure 2). When two different parameters were evaluated (the number of new ASVs increasing by <10% and <1%), it was found that at 2.8 birds (range 2–4) subsequent addition of birds resulted in less than 10% new ASV being added while 7.6 birds (range 6–10) resulted in additional samples increasing by less than 1% new ASVs. The proventriculus and gizzard required the least number of samples to reach the 1% threshold with six birds, the duodenum and jejunum required seven birds, the crop, ileum, colon, and cloacal swab need eight and finally, the ceca required 10 cecum pouches.

### Within bird cecal pairs similarity

**FIGURE 3:**
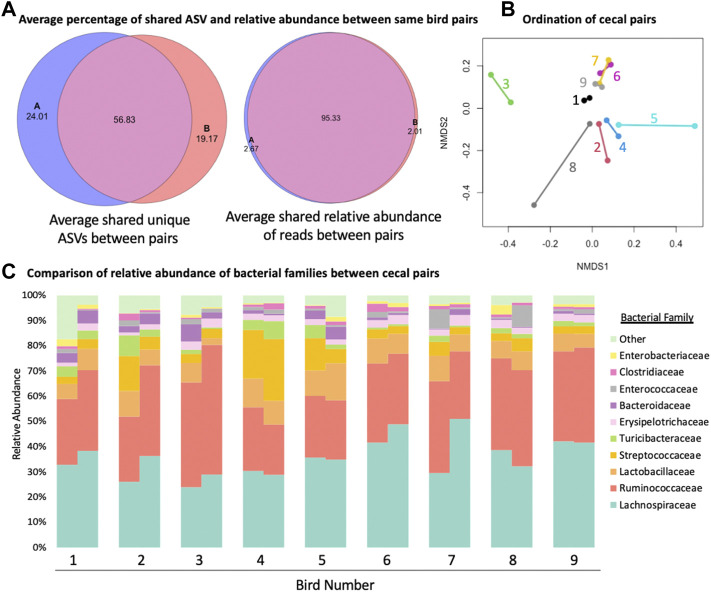
Comparison of similarity of the two cecal pouches within the same bird. **(A)** The average number of shared amplicon sequence variants (ASVs) between the two cecal pouches of the same bird and the average shared relative abundance between of reads between the two cecal pouches of the same bird. **(B)** Bet diversity of ordination of cecal pairs colored and connected by pair mate. **(C)** Staked taxonomic bar plot of relative abundance of bacterial families between cecal pairs from the same bird.

When cecal pairs among broilers were compared to each other from an ordination and composition standpoint (Figure 3), pairs were found to be similar. An average of 95.33% (range 79.33%–98.09%, Figure 3A) of the relative abundance of reads was present in both cecum within one bird. On the other hand, when unique ASVs were considered, the average number that was shared fell to 56.83% (range 23.07%–69.18%, Figure 3A). When beta diversity was considered, in most cases the nearest neighbour was the matching cecal pair mate (Figure 3B), though there was little variation among ceca pairs between birds, presumably due to all samples originating from the same flock. Finally, when family level differences were compared, some ceca pairs were more similar to their mates than other pairs (Figure 3C) but overall pairs were similar to their mate. There were not families that were consistently significantly different between cecal pairs, meaning that variation was more likely individual bird driven and not reflective of a biological difference between cecal pouches.

### Differentially enriched cecal taxa

**FIGURE 4:**
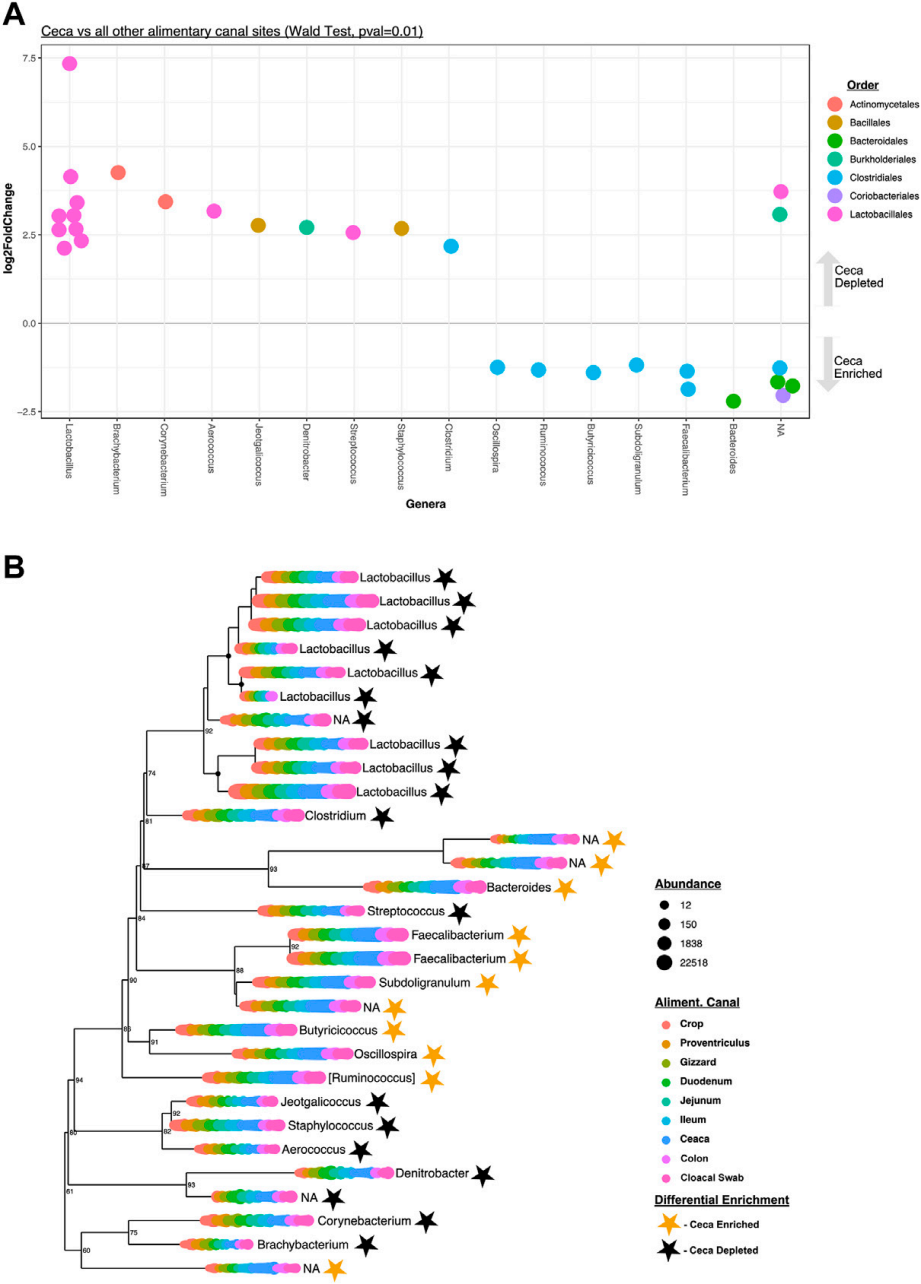
Differentially enriched cecal taxa. **(A)** Significantly (Wald Test, Pval = 0.01) differentially enriched cecal taxa (*n* = 30). Each circle represents an ASV that was either significantly enriched (negative LogFold2 values, *n* = 11) or depleted (positive LogFold2 values, *n* = 19) in cecal samples. Colored code depicts Order-level taxonomy and columnar arrangement represents Genus-level taxonomy assignments. **(B)** Phylogenetic tree of the 30 differentially enriched taxa identified depicted in **(A)**. The circles at each leaf represent the normalized abundance of each taxon at each color-coded AT site. The genus-level taxonomy assignment of each differentially enriched ASV is also shown. Stars represent the status of each taxon in the tree as either enriched (golden) or depleted (black) in cecal samples relative to all other samples.

A pooled differential abundance test model was used to compare significant (Wald Test, *p* = 0.01, Figure 4A) lineage enrichments in all cecal samples relative to all non-cecal samples. Results show 30 differentially enriched lineages, specifically, 19 and 11 lineages were cecal depleted and enriched, respectively (Figure 4B). Seven of the eleven cecal-enriched lineages belong to the bacterial order Clostridiales and include members of the following genera: Oscillospira, Ruminococcus, Butyricicoccus, Subdoligranulum, and Faecalibacterium. Other cecal-enriched lineages were classified as members of the Bacteroidales and Coriobacteriales orders. The most common cecal depleted lineages were predominantly classified as *Lactobacillus* spp. but also include members of the Actinomycetales, Bacillales, Burkholderiales, and Clostridiales orders.

### Cloacal swab representation of alimentary tract

**FIGURE 5:**
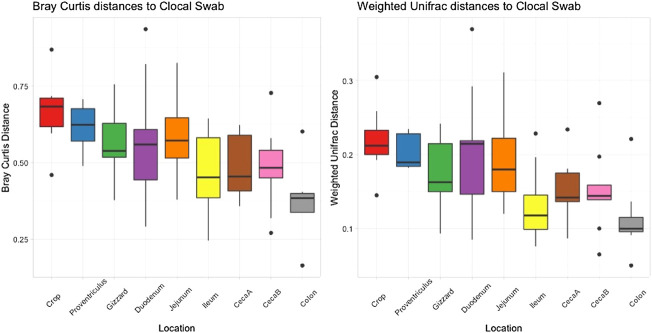
Bray Curtis dissimilarly and weighted unifrac distances between the cloacal swab and other AT location in individual birds averaged.

**FIGURE 6:**
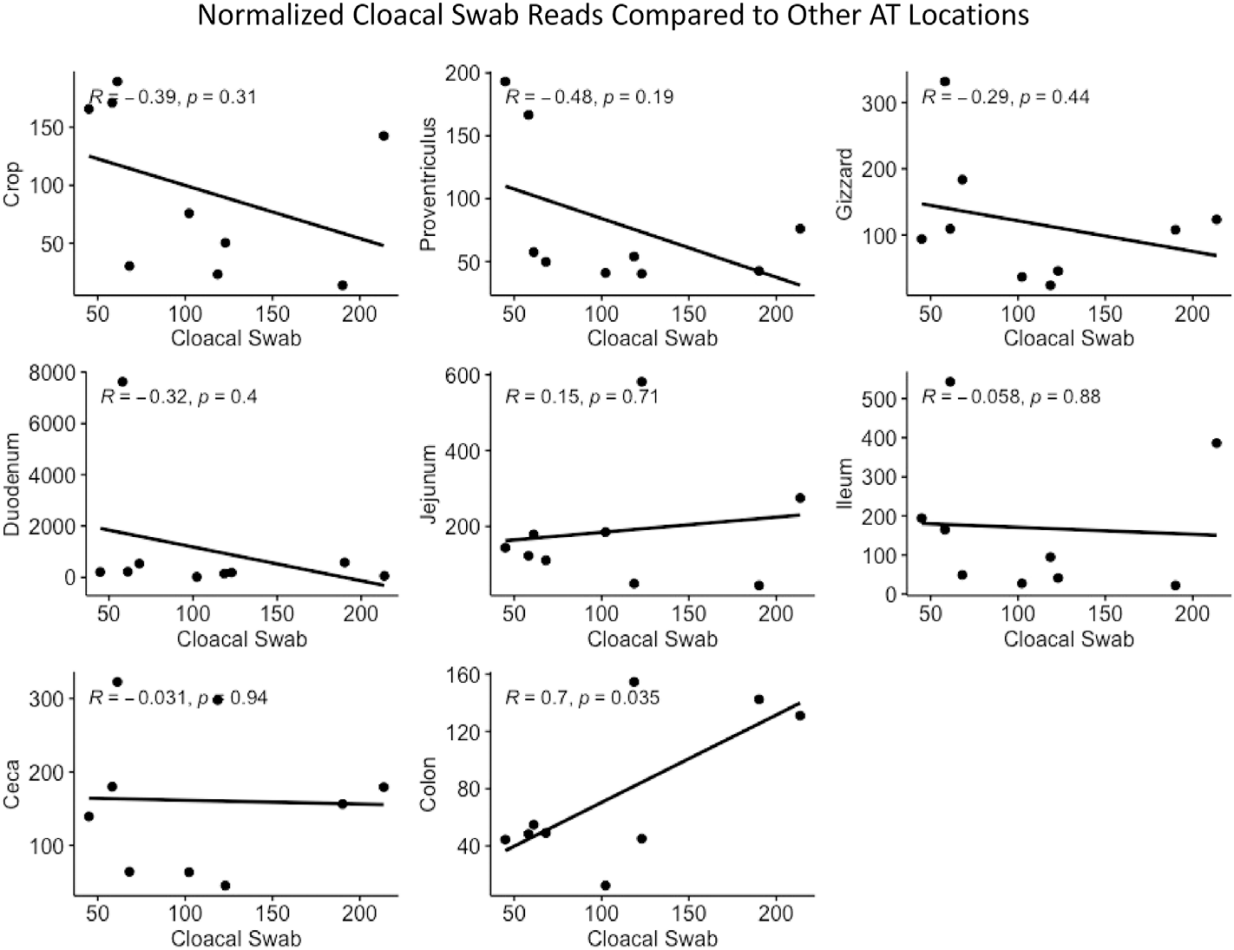
Correlation between normalized Enterobacteriaceae read numbers found in the cloacal swab and other AT locations.

Cloacal swab microbiomes were compared to nine AT location microbiomes to understand if swabs are a good predictor of the microbial community of these locations. Both Bray-Curtis dissimilarly and weighted Unifrac distances were compared at each location to the cloacal swab of the same bird. The general trend was that there was a shorter distance (more similarity) between cloacal swabs and other sampling types as the sampling was more aborad in the AT (Figure 5). Normalized *Enterobacteriaceae* read counts were compared between each AT location to the number of normalized *Enterobacteriaceae* read counts in each cloacal swab. The only significant correlation between a AT location and cloacal swabs was the adjacent colon (*p* = 0.035, *R* = 0.70, Figure 6).

**FIGURE 7:**
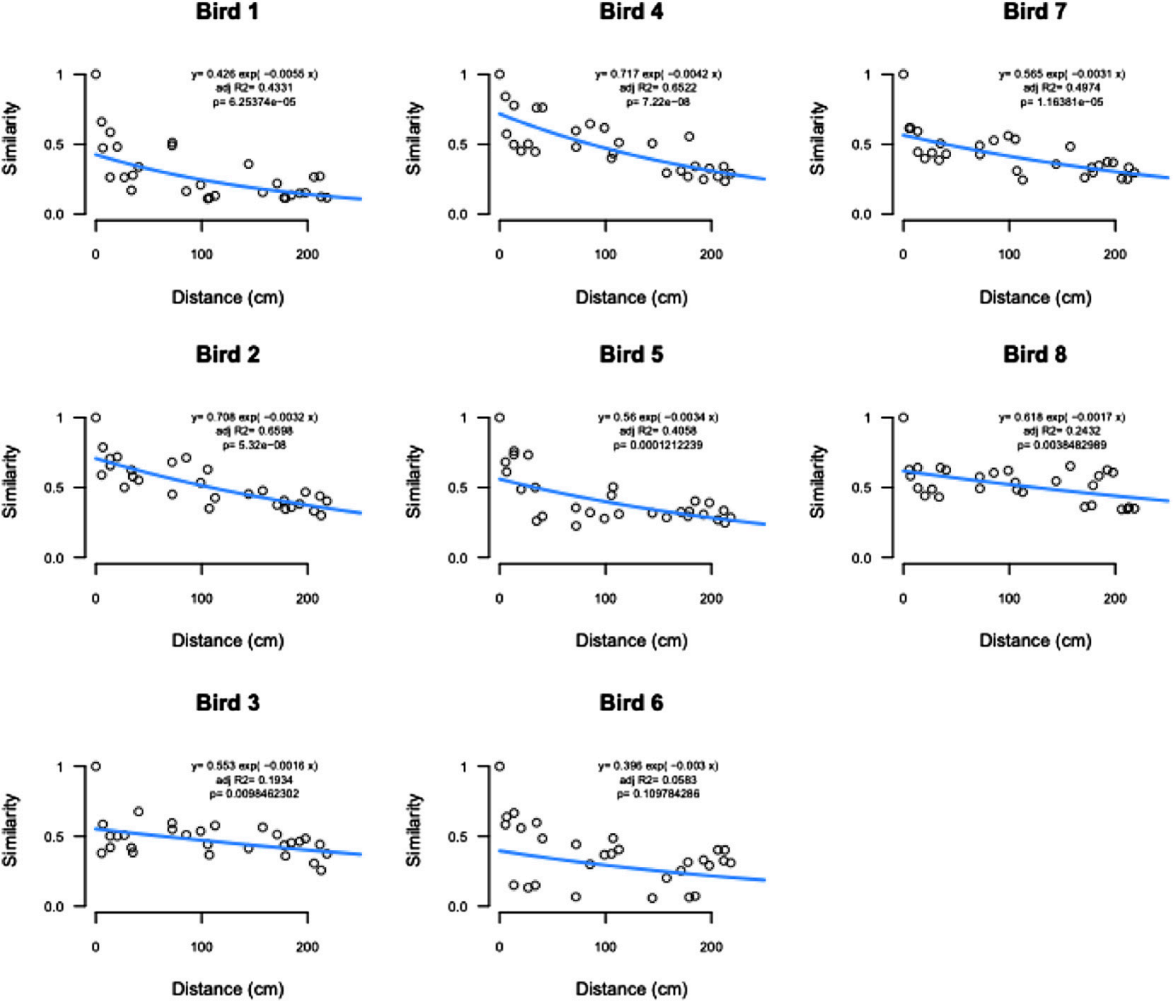
Distance decay analysis by bird showing significant relationships between community similarity and physical distance between samples for all but bird 6.

Distance-decay analysis showed significant relationships between community similarity and physical distance between samples for all but one bird (Figure 7).

## Discussion

When the microbiome of the AT described here was compared to other studies there was a high level of congruency. Past studies have also described *Firmicutes*, *Bacteroidetes*, and *Proteobacteria* as the most predominant phyla in the AT (Wei et al., 2013; Waite and Taylor, 2014). Other studies have also described different AT locations within the same birds to have distinct microbiomes (Han et al., 2016; Saxena et al., 2016) as well as reviews that have highlighted the differing predominant phyla within different AT locations (Yeoman et al., 2012; Feye et al., 2020). However, this data set is unique in the sense that there are 10 locations within the same birds that were sampled from a single flock, allowing for an understanding of the modulation of the microbiome according to location in the AT.

Past microbiome work on broilers has described differences between different flocks were smaller than the variation as a result of the age of the birds or the location sampled in the AT (Johnson et al., 2018). In terms of determining appropriate sample size within a flock, multivariant microbiome studies have proven to be more challenging, with current methods limited to specific tests, variable types, or experimental designs (Kelly et al., 2015; Mattiello et al., 2016). The data provided here estimates the amount of new data that could be gained by sampling additional birds across each AT location. Across all locations in the AT, there was a rapid decrease in the addition of new information with the addition of replicates, demonstrating a gain of no more than 10% new ASVs after the third bird and less than 1% after the eighth bird in most cases and it the case of the ceca the 10th bird. Depending on the research question, these findings can be used as a starting point to determine appropriate sample numbers within one flock. Caution must be used when classifying a collection of birds as one flock, as even small variation can result in different microbiomes. For example, even flocks that are grown in the same house but in different rooms can result in different AT microbiomes (Schokker et al., 2021).

The important biological role of the ceca from a nutritional standpoint and the ability to find many disease-causing microbes in this AT segment make it a common sampling location for broiler studies (Clench and Mathias, 1995; Clavijo and Flórez, 2018). In some studies, contents from both ceca are combined and analyzed as one sample, while in others, the pouches are considered to be replicates and different analysis are conducted on them with the bird as the experimental unit. Past work comparing the two pouches from the same bird using 16S rRNA sequencing did not find significant differences in alpha or beta diversity and noted short distances between pairs when comparing beta diversity (Stanley et al., 2015). This work agrees with the current study which demonstrated high congruency between pairs within the same bird. While the overall microbiome composition was not different between pairs, unique ASVs were found in both pouches. This paired with the fact that in terms of relative abundance more than 95% of the reads were shared, indicates that treating different cecal pouches as replicates may be appropriate with viewing the microbiome as a whole but not when looking for rare or low occurring ASV (due to the fact the while relative abundance was high, unique ASV overlap was much lower, around 56%).

Cecal-enriched lineages detected from our pooled flock comparison (e.g., *Bacteroides*, *Faecalibacterium*, *Butyricicoccus*, *Ruminococcus*, etc.) are also commonly reported from the cecal communities of commercial chickens (Ramírez et al., 2020). This suggest that the ceca are highly specialized anaerobic niches where thermodynamic constrains result in the high degree of community convergence observed across studies. The unique environment present in these organs, hosting the bulk of GI track fermentations, suggests that cecal-enriched lineages may play important roles in the avian-microbe symbiosis and, potentially, host energetic harvest. Overall, our work shows individual cecum pouch comparisons as robust community replicates to its mate and helps to define the ceca as unique and highly understudied symbiosis-relevant microbial AT environments.

The final comparisons that were made was the similarity of different AT locations to a cloacal swab and distance-decay analyses of microbial community similarity compared to physical distance between samples. We report that cloacal swabs exhibited a direct distance relationship, i.e., the more aboral samples were the more similar they were to the swab and, thus, best represent colon samples. However, we also show that this relationship may not extend to individual lineages. Cloacal swabs and feces have been used as a proxy for internal samples given their ease of access, ability to be used as a repeated measure, and non-destructive nature. There is a growing body of work on using non-lethal sampling as a proxy for sampling that requires bird euthanasia. In two studies that compared fecal to cecal swabs, Stanley et al. (2015) found that the prominent bacteria phyla in the microbiome were present in both location, with more rare OTU not found in both locations (although this comparison was not done between individual bird but instead a comparison of AT sites across a flock birds). Oakley and Kogut (2016) found distinct communities between the fecal and cecal microbiomes when compared within a bird. When cloacal swabs were evaluated, Andreani et al. (2020) found that while over 99% of the abundance of reads was captured with a cloacal swab, rare OTUs were not (but the high abundance captured in both sample types may be an effect of comparing pooled samples as opposed to individual birds). In a separate analysis in their study, they did find that the cecal samples from individual birds did have a moderate correlation to their corresponding cloacal swabs. Finally, while one study (Williams and Athrey, 2020) came to the conclusion that cloacal swabs are not an appropriate measure of the AT, this study only focused on differences in alpha and beta diversity and did not look into any correlations specific to the sample types.

Our work builds on past studies and here only compares AT locations to cloacal swabs within the same bird. Overall, it was found that the more aborad a sample was taken in the AT, the more similar it was to the cloacal swab from that bird. That is to say, the crop was the poorest approximation for the cloacal swab and the colon was the closest. Based on our findings that alpha and beta diversity were distinct between sites within AT locations and community similarity was significantly correlated with physical proximity of samples, we conclude that cloacal swabs are not a good approximation of the actual internal community at other AT locations. This conclusion is further supported by the fact that normalized read counts for *Enterobacteriaceae* between cloacal swabs and other AT locations were not significantly correlated. From a practical standpoint, this means that while cloacal and fecal swabs can be used as a loose approximation of overall microbiome shifts, it should not be used to infer changes to specific phyla—even those that are highly abundant within the microbial community.

## Data Availability

The datasets presented in this study can be found in online repositories. The names of the repository/repositories and accession number(s) can be found below: https://www.ncbi.nlm.nih.gov/, PRJNA844895.
